# Complications of lung transplantation on computed tomography: pictorial essay

**DOI:** 10.1590/0100-3984.2021.0169-en

**Published:** 2023

**Authors:** Dâmaris Versiani Caldeira Gonçalves, Murilo Marques Almeida Silva, Eduardo Kaiser Ururahy Nunes Fonseca, Izabel de Oliveira Karam, Marcelo Buarque de Gusmão Funari, Rodrigo Caruso Chate

**Affiliations:** 1 Hospital Israelita Albert Einstein, São Paulo, SP, Brazil.

**Keywords:** Lung transplantation, Postoperative complications, Multidetector computed tomography, Transplante de pulmão, Complicações pós-operatórias, Tomografia computadorizada multidetectores

## Abstract

Lung transplantation is becoming increasingly more common as an alternative treatment for end-stage lung disease. Despite advances in laboratory testing, surgical technique, and donor/recipient selection, lung transplantation is still associated with significant mortality, due to postoperative complications. This paper consists of a brief review of postoperative complications in lung transplant recipients, illustrating those complications with computed tomography images.

## Abstract (pt)

Transplantes pulmonares são procedimentos progressivamente mais realizados em todo o mundo como opção para tratamento de doenças pulmonares em estágio terminal. Apesar dos avanços laboratoriais, da técnica cirúrgica e da seleção de doadores e receptores, a mortalidade nesses procedimentos ainda é significativa, em razão de complicações típicas dos pacientes transplantados. Este trabalho consiste em uma revisão da literatura acerca do tema, ilustrando as complicações abordadas por meio de imagens de tomografia computadorizada.

## INTRODUCTION

Lung transplantation (LT), which has been performed successfully since the 1980s, now allows greater survival and quality of life for patients with various types of end-stage lung diseases^(1,2)^. In Brazil, LT is performed by six teams in three states (São Paulo, Rio Grande do Sul, and Ceará), and the number of LTs performed in the country increased by 50% between 2013 and 2018^(3)^.

Currently, bilateral (sequential) LT is performed more often than is unilateral LT, because the former is associated with better survival^(1,4)^. However, mortality increases over time after the procedure. Most deaths occurring in the first six months post-LT are due to infectious complications, whereas chronic graft dysfunction (CGD) is typically implicated in deaths occurring thereafter^(1,5-8)^.

Despite advances in surgical techniques and immunosuppressive treatments, post-LT complications are still common. Therefore, patients undergoing LT require periodic clinical follow-up, including pulmonary function tests and imaging examinations^(4)^ .The imaging findings after LT are the focus of this essay, in which we analyze those obtained in the immediate postoperative period (the first 24 h after the procedure), the intermediate postoperative period (between 24 h and two months after the procedure), and the late postoperative period (more than two months after the procedure).

## IMMEDIATE POSTOPERATIVE PERIOD

Among the complications of LT that occur in the immediate postoperative period are the formation of acute pleural collections (pneumothorax, hemothorax, pleural effusion, or empyema), hyperacute rejection, and lung size mismatch^(1)^.

**Figure 1:**
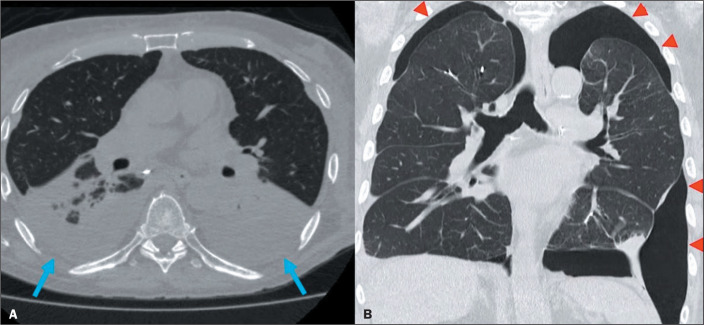
CT scans showing acute pleural complications after LT. A: Moderate bilateral pleural effusion (arrows). B: Bilateral pneumothorax (arrowheads).

Pleural complications are quite common in the immediate postoperative period after LT. Although practically all recipients develop pleural effusion (Figure 1A), it tends to resolve within approximately two weeks. Progression to empyema should be suspected if the collection persists for more than a month^(1,4)^. Another common pleural complication is pneumothorax^(1,2,4,5)^, as illustrated in Figure 1B.

Hyperacute rejection after LT is a fulminant immune-mediated reaction to antigens present in the lung graft. Although this condition is now rare, thanks to advances in pretransplant testing, its occurrence is associated with high mortality. On imaging, hyperacute rejection manifests as diffuse pulmonary opacities and signs of pulmonary edema^(6)^.

It is known that the use of lungs that are too large or too small for the recipient (lung size mismatch) can compromise the results of LT. If the lung is too large, the recipient will experience ventilatory restriction, whereas the transplantation of a lung that is too small will result in persistent pleural collections and pulmonary hyperinflation^(1,2,4,5)^.

## INTERMEDIATE POSTOPERATIVE PERIOD

Among the complications of LT that occur in the intermediate postoperative period is primary graft dysfunction-previously known as ischemia-reperfusion injury, edema, or reimplantation response-which is characterized by hypoxia and pulmonary opacities of noncardiogenic origin appearing within the first 24 h after LT, peaking at 72 h after LT and typically resolving spontaneously after post-LT day 10^(1,4,6)^. In LT recipients with primary graft dysfunction, thickening of the interlobular septa and perihilar parenchymal opacities are observed, usually distributed throughout the lower lung fields. The persistence of primary graft dysfunction after post-LT day 10 or marked radiological improvement after corticosteroid treatment should raise the suspicion of acute rejection^(6)^.

**Figure 2:**
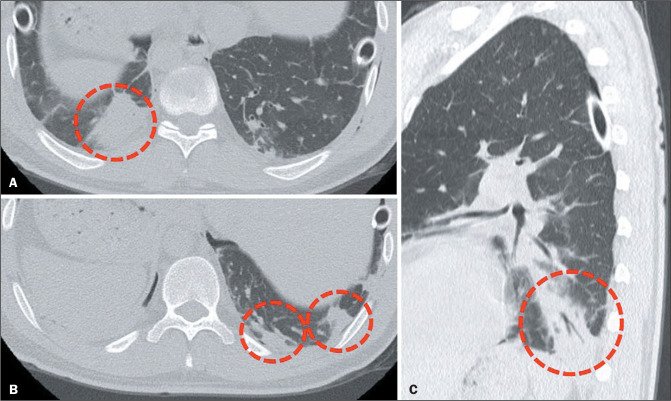
CT scans showing postoperative pneumonia in an LT recipient. Note the multiple foci of consolidation with air bronchograms in the lower lobes (circles).

Special attention should be paid to pulmonary infections (Figure 2), which constitute the main cause of mortality in the intermediate postoperative period after LT^(1)^. Patients undergoing LT are more susceptible to pneumonia in the transplanted lung because of a number of factors, including postoperative impairment of the cough reflex, of mucociliary function, and of lymphatic drainage, as well as the immunosuppressive regimen^(1,2,7)^. More than half of all post-LT lung infections are caused by bacteria (especially *Staphylococcus aureus* and *Pseudomonas aeruginosa*), although other pathogens, such as viruses (e.g., cytomegalovirus) and fungi (e.g., *Aspergillus* sp.), are also relevant^(1,4,7)^. The imaging manifestations of such infections are varied, and it is rarely possible to determine the pathogen responsible on the basis of the computed tomography (CT) findings alone^(8)^. It should be borne in mind that infectious complications are not restricted to the intermediate postoperative period, often extending into the late postoperative period, viral infections being the most prevalent, especially after post-LT month six. More rarely, infections caused by other pathogens, such as atypical mycobacteria, can occur in the late postoperative period^(8,9)^.

**Figure 3:**
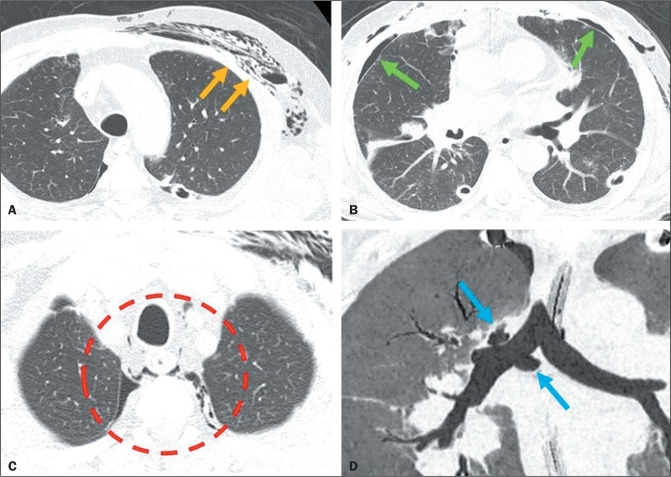
Signs of post-LT air leakage on CT. A: Emphysema in the left chest wall (arrows). B: Small bilateral pneumothorax (arrows). C: Small pneumomediastinum (circle). D: Bronchial dehiscence (arrows indicate discontinuity of the bronchial wall).

Bronchial dehiscence is another complication that manifests in the intermediate postoperative period after LT (Figure 3), affecting up to 10% of lung transplant recipients^(7)^. Although bronchoscopy is the gold standard for the diagnosis of bronchial dehiscence, imaging examinations may indirectly suggest it by demonstrating *de novo* or persistent pneumothorax, or even signs of pneumomediastinum with subcutaneous emphysema^(5,7)^.

**Figure 4:**
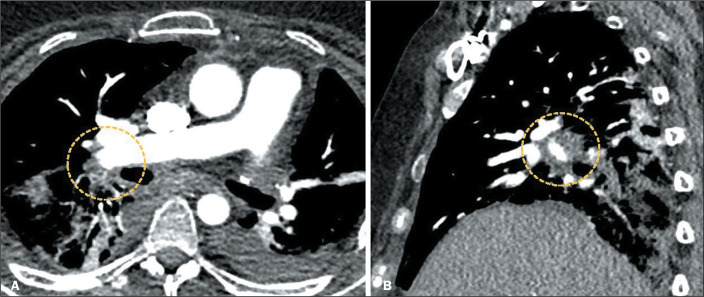
Pulmonary thromboembolism. CT angiography of the chest showing filling defects in the right pulmonary artery (circles).

Pulmonary thromboembolism, which is often associated with deep vein thrombosis, is still a common complication the intermediate postoperative period after LT^(7)^. The use of CT angiography of the chest facilitates the diagnosis of pulmonary thromboembolism (Figure 4), allowing the identification of filling defects and dilatation of the pulmonary arteries, as well as alterations secondary to parenchymal infarction^(1,4,7)^.

## LATE POSTOPERATIVE PERIOD

**Figure 5:**
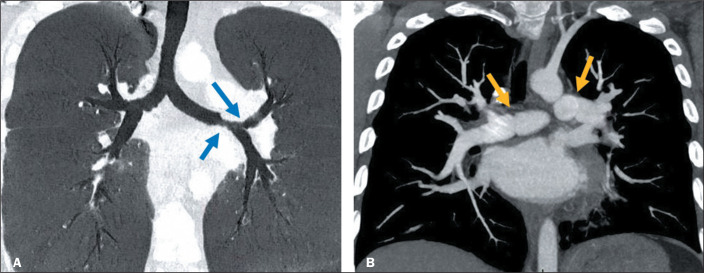
Bronchial and vascular strictures. A: Minimum-intensity projection reconstruction of a coronal CT scan, showing focal narrowing in the left main bronchus and at the origin of the left upper bronchus (arrows). B: CT angiography of the chest showing mild focal stenosis in the pulmonary arteries (arrows).

Late complications after LT include bronchial and vascular stenosis at the graft anastomosis sites (Figures 5A and 5B, respectively). Bronchial stenosis, defined as fixed narrowing of the bronchial lumen, is the most common airway complication among lung transplant recipients, reportedly occurring in up to 24% of cases^(2,4,5,7)^. Factors that can increase the risk of stenosis include infections and graft rejection, as well as the bronchial anastomosis technique employed^(4,7)^. Vascular stenosis, which is less common, can result in arterial hypertension and persistent hypoxemia due to involvement of the pulmonary arteries^(7)^. Chest CT angiography can help establish a diagnosis of vascular stenosis, allowing the point of narrowing, as well as any arterial tortuosity, to be identified^(4,7)^.

**Figure 6:**
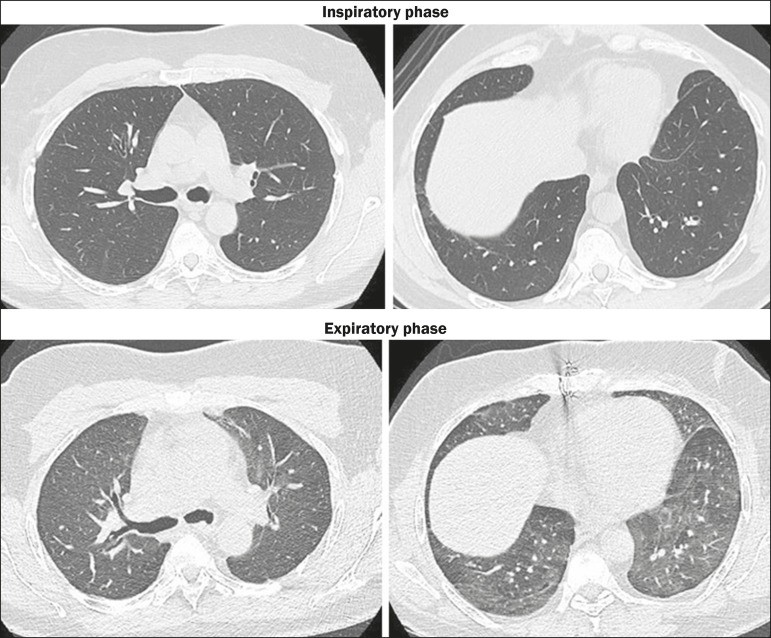
CGD at five years after LT. In the inspiratory phase, the lungs appear normal, whereas signs of air trapping, predominantly in the upper lung fields, are apparent in the expiratory phase.

The late complication that has the greatest impact on long-term survival after LT is CGD, which encompasses the phenotypes of bronchiolitis obliterans syndrome (BOS) and restrictive allograft syndrome^(10)^. The diagnosis of CGD fundamentally consists in the detection of an irreversible, > 20% reduction in the forced expiratory volume in one second in relation to the baseline value, and the development of CGD is associated with imbalances among the autoimmune response, the inflammatory response, and tissue repair processes^(10)^. The most common form of CGD is BOS, which is seen in nearly 50% of patients within the first five years after LT. On CT, the presentation of BOS varies according to the severity of the condition, ranging from initially normal findings to areas of air trapping as the syndrome progresses (Figure 6). Restrictive allograft syndrome has a worse prognosis; its most striking feature is architectural distortion, especially in the upper lobes^(7,10)^. Also noteworthy is azithromycin-reversible lung allograft dysfunction, the CT presentation of which is similar to that of BOS, although its reversibility with the use of azithromycin raises debate in the literature regarding its inclusion in the CGD spectrum^(7,10)^. Transplant recipients with CGD may or may not develop organizing pneumonia, which is seen in nearly 30% of LT recipients, in whom the CT pattern is similar to that seen in nontransplant patients with organizing pneumonia^(1)^.

**Figure 7:**
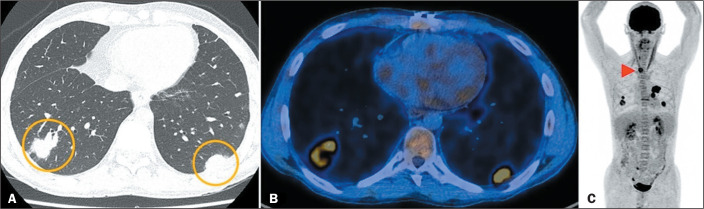
PTLD. A: CT scan showing sparse rounded parenchymal opacities interspersed with air bronchograms (circles). B,C: ^18^F-fluorodeoxyglucose positron emission tomography/CT scan showing uptake of the tracer by lung lesions, as well as by an enlarged right paratracheal lymph node (arrowhead).

Because the immunosuppressed state increases the risk of neoplasia^(11)^, the development of malignancy as a late complication of LT also merits attention. The most commonly reported malignancy is post-transplant lymphoproliferative disorder (PTLD), which is often associated with Epstein-Barr virus infection, and the relative risk of its development in the context of LT is 58.6^(11,12)^. There have been reports of early-onset PTLD, occurring within the first month after LT, which can present as multiple pulmonary nodules, consolidations, interlobular septal thickening, pleural effusion, or mediastinal lymph node enlargement^(1,2)^. As illustrated in Figure 7, ^18^F-fluorodeoxyglucose positron emission tomography/CT has a sensitivity of 85% and a specificity of 90% for the diagnosis of PTLD^(13)^. However, post-LT malignancies are not restricted to PTLD, a higher-than-average incidence of skin cancer (especially nonmelanoma skin cancer) and of cancer affecting other organs (especially the lungs and gastrointestinal tract) having been reported among LT recipients^(11,14)^.

## CONCLUSION

**Figure 8:**
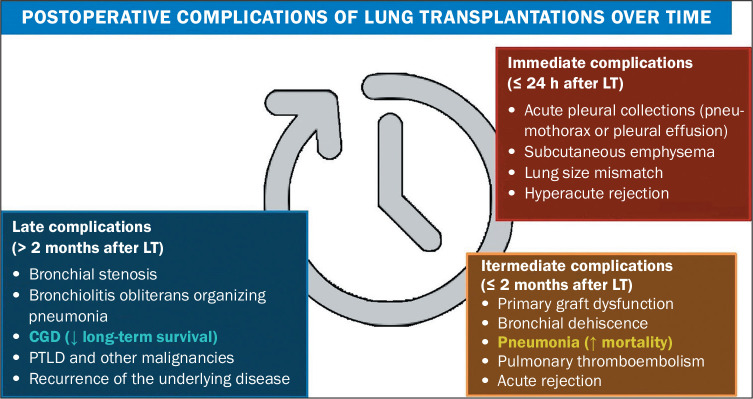
Summary of the main postoperative complications of LT over time.

Complications related to LT affect the recipients at different time points after transplantation. For example, pleural collections and acute rejection manifest immediately after the procedure, whereas vascular and airway complications arise in the intermediate or late postoperative periods after LT, as do respiratory infections and recurrence of the underlying lung disease. Malignancy and CGD are more common in the late postoperative period. The temporal evolution of post-LT complications is summarized in Figure 8. Nevertheless, it should be borne in mind that the primary lung disease that motivated the transplant can recur at any time after LT^(1)^. Given the progressive increase in the number of LTs performed in Brazil, radiologists should expand their knowledge of the potential postoperative complications, which will favor the early recognition of such complications, thus reducing postoperative mortality.
